# Polyoxometalate/Ionic Liquid Desulfurization System for Hydrogen Sulfide Removal from High-Temperature Gas Stream

**DOI:** 10.3390/molecules27196723

**Published:** 2022-10-09

**Authors:** Junpeng Li, Rui Wang

**Affiliations:** School of Environmental Science and Engineering, Shandong University, No. 27 Shanda South Road, Jinan 250199, China

**Keywords:** polyoxometalate, H_2_S removal, ionic liquid, sulfur recovery

## Abstract

The temperature of industrial gas containing harmful H_2_S can reach hundreds of degrees. However, few processes can be used directly for H_2_S removal from industrial high-temperature gas. In this work, three polyoxometalates with different central atoms ((n-Bu_4_N)_3_VMo_12_O_40_, (n-Bu_4_N)_3_PMo_12_O_40_, and (n-Bu_4_N)_4_[α-SiMo_12_O_40_]) were synthesized and dissolved in four ionic liquids (Bmim]Cl, [Bmim]HCO_3_, [Bmim]Mes, or [Bmim]OAc) for H_2_S removal from high-temperature (90–180 °C) gases. The result showed that (n-Bu_4_N)_3_VMo_12_O_40_/[Bmim]OAc exhibited the optimal desulfurization performance, maintaining more than 98.6% desulfurization efficiency within 10 h. The reacted desulfurization solution can be regenerated by blowing air. FT-IR and XPS results show that both the central atom V and the coordination atom Mo of the polyoxometalate are involved in the oxidation of H_2_S; after the regeneration by introducing air, V(+IV) and Mo(+IV) recovered to V(+V) and Mo(+VI), respectively. Our research shows that (n-Bu_4_N)_3_VMo_12_O_40_/[Bmim]OAc is an efficient, easy-to-regenerate, and suitable high-temperature gas desulfurization solution.

## 1. Introduction

Hydrogen sulfide (H_2_S) is a toxic and harmful gas widely present in natural gas, refinery waste gas, coke oven gas, and syngas [1,2]. It not only poses a threat to human health, but also causes corrosion of production equipment and pipelines and poisoning of catalysts [3,4]. Therefore, it must be removed and transformed. The process of H_2_S removal is divided into the dry process and the wet process. The dry process mainly includes the molecular sieve method, activated carbon method, zinc oxide method, iron oxide method, etc. [2]. These desulfurization processes are characterized by high desulfurization precision, simple equipment, and convenient operation, but low desulfurization load and difficult regeneration. The wet process has the advantage of a high desulfurization load. Among them, the alcohol amine solution method is the most widely used wet desulfurization process [5]. However, this organic solvent and other solvents (usually water) have a very low applicable temperature and are difficult to meet the requirements of high-temperature desulfurization [4]. The temperature of some industrial waste gas can reach hundreds of degrees, and it must be cooled before entering the traditional wet desulfurization process, which increases the investment cost [2]. Therefore, it is of great significance to develop a desulfurization system with high-temperature resistance, easy regeneration, and high efficiency for the H_2_S removal from high-temperature gas.

Ionic liquids are salts that are liquid at low temperature (<100 °C) which represent a class of solvents with nonmolecular, ionic character [6]. Wang et al. [7,8] reported a novel supported ionic liquid phase Cs-based system for the conversion of 1,1,2-Trichloroethane to 1,1-Dichloroethylene. The results showed that the addition of ionic liquids could significantly improve the dispersion of Cs species in the preparation and performance evaluation of the catalyst, thereby significantly improving catalytic activity. In addition, ionic liquids change the adsorption behavior of the product, thereby improving the selectivity of the product 1,1-Dichloroethylene. Ionic liquid has extremely low vapor pressure, a wide liquid phase range, and good stability at high temperature [9]. These characteristics make ionic liquids an ideal solvent for high-temperature wet desulfurization. At present, most of the research on ionic liquids in the field of H_2_S absorption is concentrated under high-pressure static conditions [10,11,12]. There are few studies on H_2_S removal under dynamic conditions of atmospheric pressure and high temperature. The H_2_S removal by using ionic liquid mainly relies on the physical absorption or alkalinity of ionic liquid, and the desulfurization performance is very limited [12,13]. Polyoxometalates are a general term for a class of oxygen-containing polybasic acids with elements such as P, Si, Ge, and S as the central atom and elements such as Mo, W, and V as the coordination atom, bridged with oxygen atoms. Polyoxometalates have not only unique redox and catalytic properties, but also good thermal stability. Combining polyoxometalates and ionic liquids may meet the needs of high-temperature wet oxidative desulfurization. The redox properties of polyoxometalates are crucial for the desulfurization performance of polyoxometalate/ionic liquid desulfurization systems [14]. The central atoms, coordination atoms, and substitution atoms of polyoxometalates all influence the redox properties of polyoxometalates. Ma et al. [15] studied the removal performance of phosphomolybdovanadate (H_6+n_[P_2_Mo_18−n_V_n_O_62_] (n = 1, 2, 3, or 4)) with different numbers of vanadium atom substitutions for H_2_S removal and found that the monovanadium-substituted phosphomolybdovanadate H_7_[P_2_Mo_17_VO_62_] aqueous solution showed the best desulfurization performance. Liu et al. [2] studied the H_2_S removal performance of Mo-substituted phosphotungstic acid (PW_12-x_Mo_x_O_40_, x = 0, 1, 3, 6, 9, 12) and V-substituted phosphomolybdic acid (PMo_12−x_V_x_O_40_, x = 0, 1, 2, 3, 6). The results show that the desulfurization performance of PW_12−x_Mo_x_O_40_ solution is enhanced with the increase in the number of substitution atoms Mo, indicating that Mo atoms are more suitable for H_2_S removal than W atoms. Among PMo_12−x_V_x_O_40_, PMo_10_V_2_ solution exhibits the best desulfurization performance. However, the effect of the central atom species on the H_2_S removal performance is unknown.

The purpose of this work is to develop a desulfurization system that is highly efficient, easy to regenerate, and suitable for high-temperature gas. Polyoxometalates with different central atoms were synthesized and dissolved in different ionic liquids to obtain polyoxometalates/ionic liquid desulfurization solution. The effects of various factors on the desulfurization performance were investigated under normal pressure and high-temperature conditions (90–180 °C). After the reaction, the air was pumped into the desulfurization system to realize the regeneration of the desulfurization solution. The desulfurization solution and products were analyzed by FT-IR, TGA-DSC, XRD, and XPS. The results show that the desulfurization products are elemental sulfur and sulfate, and the desulfurization system can realize multiple cycles of desulfurization at high temperature.

## 2. Results and Discussion

### 2.1. Polyoxometalate and Ionic Liquid Characterization

**Figure 1:**
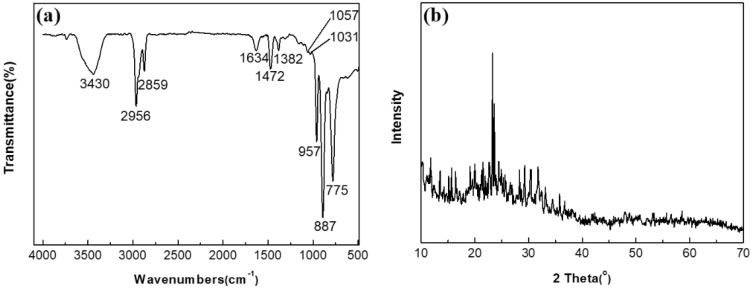
FT-IR spectra (**a**) and XRD pattern (**b**) of (n-Bu_4_N)_3_VMo_12_O_40_.

**Figure 2:**
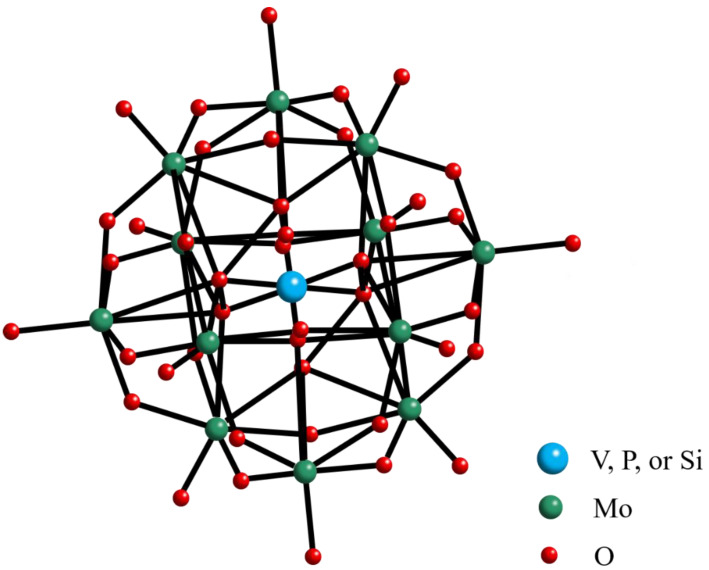
Structural diagram of polyoxometalate anion XMo_12_O_40_ (X = V, P, or Si).

**Figure 3:**
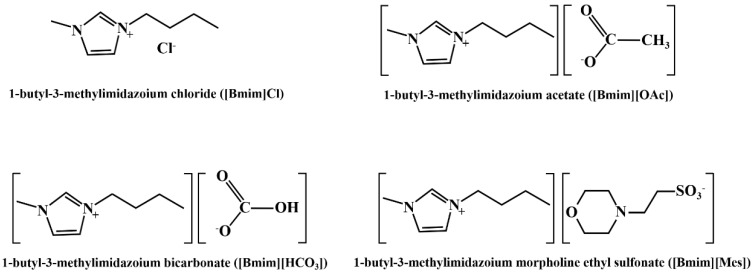
Molecular structure diagram of four ionic liquids.

The FT-IR spectrum of (n-Bu_4_N)_3_VMo_12_O_40_ is displayed in Figure 1a. The peak at 957 cm^−1^ is the stretching vibration peak of the Mo=O_d_ bond, the peak at 887 cm^−1^ is attributed to the stretching vibration peak of V-O bond, and the peak at 775 cm^−1^ is the stretching vibration of Mo-O_c_-Mo bond peak [16,17,18]. Figure 1b is the XRD pattern of (n-Bu_4_N)_3_VMo_12_O_40_. The diffraction peaks at 11.7°, 15.6°, 23.8°, and 31.7° are the characteristic peaks of Keggin polyoxometalates [19,20,21]. The FT-IR and XRD spectra revealed that (n-Bu_4_N)_3_VMo_12_O_40_ was successfully synthesized. The structure diagram of polyoxometalate anion XMo_12_O_40_ (X = V, P, or Si) and the molecular structure diagram of ionic liquids are shown in Figure 2 and Figure 3.

**Figure 4:**
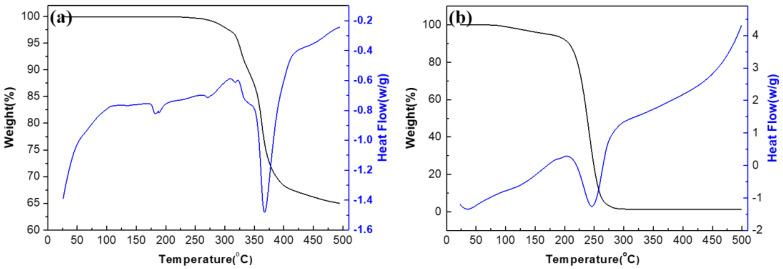
TGA-DSC curves of (**a**) (n-Bu_4_N)_3_VMo_12_O_40_ and (**b**) [Bmim]OAc.

Figure 4a shows the TGA-DSC curve of (n-Bu_4_N)_3_VMo_12_O_40_. There is only a 0.32% mass loss between 25–250 °C, which is caused by the evaporation of a small amount of water. Between 300–400 °C, the thermogravimetric curve decreased rapidly, the mass loss of the sample was 31.64%, and the DSC curve showed an obvious endothermic peak, indicating that (n-Bu_4_N)_3_VMo_12_O_40_ decomposed in this temperature range. However, (n-Bu_4_N)_3_VMo_12_O_40_ is completely stable at the highest operating temperature of 180 °C in this experiment. Figure 4b shows the TGA-DSC curve of ionic liquid [Bmim]OAc. As can be seen from the figure, the weight loss of the sample is 5.4% between 25–180 °C, which is mainly due to the evaporation of water. Between 200–250 °C, the TGA curve decreased rapidly, the sample weight loss was 67.5% in this range, and the DSC curve showed an endothermic peak in this range, indicating that [Bmim]OAc was decomposed in this temperature range. The TGA-DSC curve shows that the thermal stability of [Bmim]OAc also meets the requirements of high-temperature (<180 °C) gas desulfurization.

### 2.2. Desulphurization Performance

**Figure 5:**
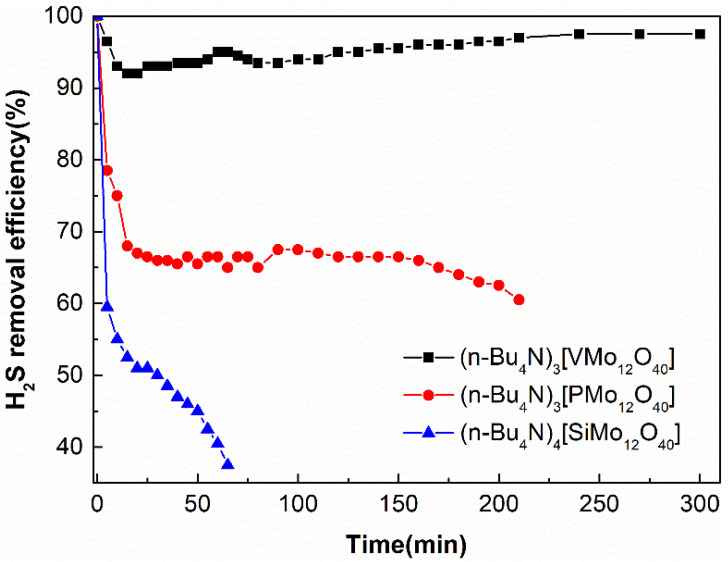
The effects of central atom on H_2_S removal.

The central atom of the polyoxometalate (i.e., heteroatom, which can be S^6+^, V^5+^, P^5+^, As^5+^, Ge^4+^, Si^4+^, B^3+^, etc.) affects its redox properties by changing the charge number of the heteropoly anion. As the number of charges increases, the oxidation potential of polyoxometalates decreases, for example, SMo_12_O_40_^2−^ > PMo_12_O_40_^3−^ > SiMo_12_O_40_^4−^, PW_12_O_40_^3−^ > SiW_12_O_40_^4−^ ≈ GeW_12_O_40_^4−^ > BW_12_O_40_^5−^ [22,23]. As shown in Figure 5, we used [Bmim]Cl as a solvent to investigate the effect of central atom species on desulfurization performance. It can be seen that the central atom has a great influence on the desulfurization performance of polyoxometalates. The desulfurization properties of polyoxometalates with different central atoms are V > P > Si, which matches the theoretical oxidation potential polyoxometalates (VMo_12_O_40_^3−^ > PMo_12_O_40_^3−^ > SiMo_12_O_40_^4−^). The (n-Bu_4_N)_3_VMo_12_O_40_/[Bmim]Cl solution can maintain a desulfurization rate of more than 92% within 300 min. Therefore, (n-Bu_4_N)_3_VMo_12_O_40_ was selected as the solute for the next experiment.

**Figure 6:**
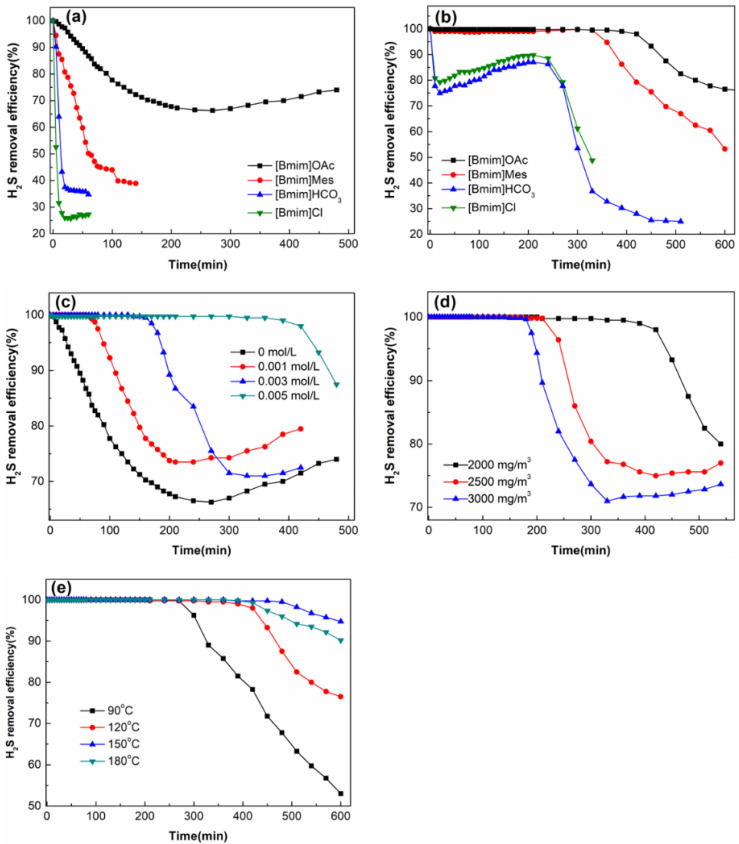
H_2_S removal performance of (**a**) single ionic liquids and (**b**) (n-Bu_4_N)_3_VMo_12_O_40_ in different ionic liquids; effects of (**c**) polyoxometalate concentration, (**d**) H_2_S concentration, and (**e**) reaction temperature on H_2_S removal.

The absorption of H_2_S by ionic liquids may affect the oxidative removal of H_2_S by polyoxometalates. For dynamic removal of H_2_S, the better the absorption of H_2_S by the ionic liquid, the more reaction time the polyoxometalates dissolved in the ionic liquid have to oxidize the dissolved H_2_S. Factors such as the type of anion and cation, density, alkalinity, and hydrogen bond of the ionic liquid can affect the absorption performance of H_2_S by the ionic liquid [24]. Jalili et al. [13,24,25] investigated the solubility of H_2_S in ionic liquids formed by the cation [Hoemin] and the anions Tf_2_N^−^, OTF^−^, PF_6_^−^, and BF_4_^−^. They found that the order of the solubility of H_2_S in these four ionic liquids was opposite to the order of the density of the ionic liquids. As the density of the ionic liquid increases, the volume of the ionic liquid (or its free volume) decreases, thus reducing the potential for acidic gases such as H_2_S to come into contact with the ionic liquid, and so the gas solubility decreases. Huang et al. [26] studied the H_2_S absorption in imidazole ionic liquids with carboxylate as an anion, such as lactate (Lac), acetate (Ac), and propionate (Pro). The carboxylate is somewhat basic and forms a strong acid-base and hydrogen bond with H atoms of H_2_S, so this ionic liquid has a high absorption capacity for H_2_S. Under the same conditions, the order of absorption of H_2_S by ionic liquids with anions is Lac < Ac < Pro, which is consistent with the basic order of the anions. In the present work, we investigated the absorption performance of four ionic liquids by themselves (without polyoxometalates) for H_2_S, as shown in Figure 6a. The order of absorption of H_2_S by ionic liquids is [Bmim]Cl < [Bmim]HCO_3_ < [Bmim]Mes < [Bmim]OAc. The pKa values for acetic acid (HOAc), morpholinoethanesulfonic acid (Mes), and carbonic acid (H_2_CO_3_) are 4.74, 6.1, and 6.37, respectively. According to the conjugate acid-base theory, the order of alkalinity of these three anions is OAc^−^ < Mes^−^ < HCO_3_^−^. It does not appear that the hydrogen bonding theory can be fully used to explain the absorption of H_2_S, as the H_2_S absorption performance does not coincide with the order of alkalinity of the anions. The underlying mechanisms affecting the absorption performance of these four ionic liquids will need to be investigated in future work. Figure 6b shows the H_2_S removal performance of (n-Bu_4_N)_3_VMo_12_O_40_ dissolved in different ionic liquids. H_2_S removal performance improves in the following order: [Bmim]HCO_3_ ≈ [Bmim]Cl < [Bmim]Mes < [Bmim]OAc. (n-Bu_4_N)_3_VMo_12_O_40_/[Bmim]OAc solution exhibited the best desulfurization performance, maintaining more than 98% desulfurization performance within 420 min. Comparing Figure 6a,b, it is found that the H_2_S removal performance of the polyoxometalate/ionic liquid is significantly improved after adding the polyoxometalate, indicating that the polyoxometalate (n-Bu_4_N)_3_VMo_12_O_40_ plays the main role in desulfurization. At the same time, it can also be found that the order of the H_2_S removal performance of the (n-Bu_4_N)_3_VMo_12_O_40_/ionic liquid is very similar to that of the single ionic liquid. The difference in the H_2_S removal performance of the polyoxometalate dissolved in different ionic liquids may be caused by the difference in the solubility of H_2_S in ionic liquids. [Bmim]OAc was selected as the solvent for the polyoxometalate for the next experiment. The effect of polyoxometalate concentration on H_2_S removal performance is shown in Figure 6c. When the concentration of (n-Bu_4_N)_3_VMo_12_O_40_ was 0.005 mol/L, the desulfurization solution could maintain a desulfurization efficiency of more than 99% within 390 min, and the desulfurization efficiency gradually decreased after 390 min. The desulfurization efficiency increases gradually with the increase in the concentration of polyoxometalates. Comparing the desulfurization curves of 0 and 0.005 mol/L polyoxometalates, it can be seen that the polyoxometalate plays a major role in the desulfurization process. In the following experiments, the concentration of the polyoxometalate was chosen to be 0.005 mol/L. Figure 6d shows the effect of H_2_S concentration on desulfurization performance. It can be seen from the figure that the removal efficiency of H_2_S decreases with the increase in H_2_S concentration. Within 180 min, the desulfurization solution can maintain 100% removal efficiency under different H_2_S concentrations because the amount of polyoxometalate is sufficient to oxidize H_2_S within this period. After 180 min, the desulfurization efficiency began to decrease. The higher the H_2_S concentration, the faster the desulfurization efficiency decreased. This is because the higher the amount of H_2_S introduced, the faster the consumption of the polyoxometalate. In short, the lower the H_2_S concentration, the better the H_2_S removal efficiency. The effect of reaction temperature on H_2_S removal performance is shown in Figure 6e. When the reaction temperature increased from 90 °C to 150 °C, the desulfurization efficiency increased accordingly. When the reaction temperature continued to increase to 180 °C, the desulfurization efficiency decreased. The temperature has two effects on H_2_S removal. On the one hand, increasing the temperature is beneficial to reducing the viscosity of the ionic liquid and thus speeding up the mass transfer rate, and at the same time, the high temperature is also beneficial to speeding up the reaction rate. On the other hand, since the reaction between the polyoxometalate and H_2_S is exothermic, high temperature is not conducive to the progress of the reaction [5]. The two influences jointly determine the H_2_S removal efficiency. In this experiment, the desulfurization solution showed the best desulfurization performance at 150 °C. Therefore, 150 °C was chosen as the optimal reaction temperature.

### 2.3. Regeneration Performance

**Figure 7:**
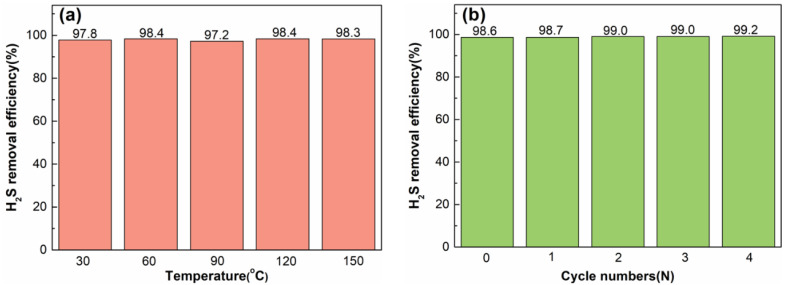
(**a**) The effect of regeneration temperature on regeneration performance; (**b**) multiple regeneration performance.

The easy regeneration of the desulfurization solution is very important for the desulfurization performance and economic cost of the whole desulfurization process. In this work, the regeneration of the desulfurization solution can be realized only by introducing air into the desulfurization solution after the desulfurization is completed. The effects of regeneration temperature and multiple desulfurization cycles on regeneration performance were investigated. The effect of regeneration temperature on the regeneration performance of the desulfurization system is shown in Figure 7a. The vertical axis represents the percentage of the amount of H_2_S removed to the total amount of H_2_S introduced within 10 h. It can be seen from the figure that the regeneration temperature has little effect on the regeneration performance. At 30 °C, the desulfurization solution can be easily regenerated, and the desulfurization efficiency after regeneration can reach 97.8%. When the regeneration temperature is 60 °C and 120 °C, the regeneration performance reaches the maximum value of 98.4%. From the perspective of energy saving, the lower the regeneration temperature, the better the energy-saving effect. Therefore, 60 °C was chosen as the regeneration temperature in this experiment. The multiple regeneration performance of (n-Bu_4_N)_3_VMo_12_O_40_/[Bmim]OAc is shown in Figure 7b. After four cycles of use, the desulfurization efficiency within 10 h can be maintained above 98.6%. It can be seen that (n-Bu_4_N)_3_VMo_12_O_40_/[Bmim]OAc is an efficient and easily regenerated desulfurization solution.

### 2.4. Desulphurisation Product

**Figure 8:**
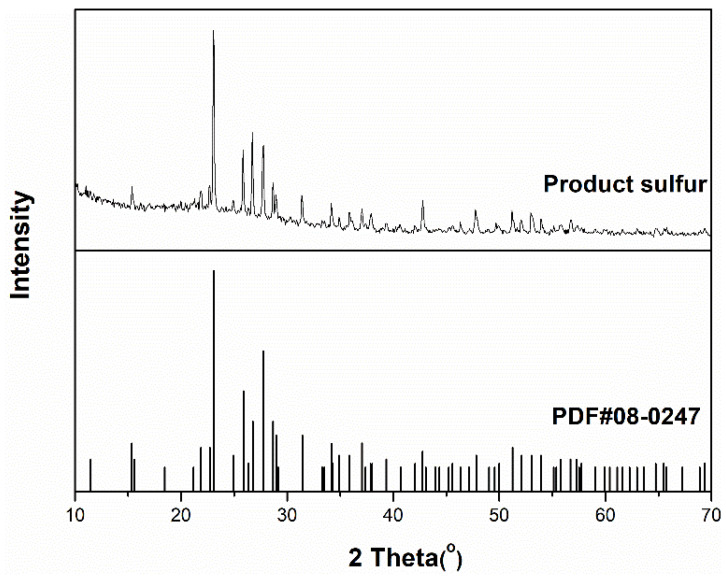
XRD spectra of desulphurization product and sulfur standard card.

As shown in Figure 8, the XRD pattern of the obtained desulfurization product is in good agreement with the sulfur standard card (PDF#08-0247), indicating that the obtained desulfurization product is elemental sulfur. The characteristic peaks at 23.04°, 25.84°, and 27.76° indicate that the obtained elemental sulfur is orthorhombic. This result indicates that the desulfurization solution (n-Bu_4_N)_3_VMo_12_O_40_/[Bmim]OAc converts toxic H_2_S into an economically valuable elemental sulfur resource.

### 2.5. Mechanism

**Figure 9:**
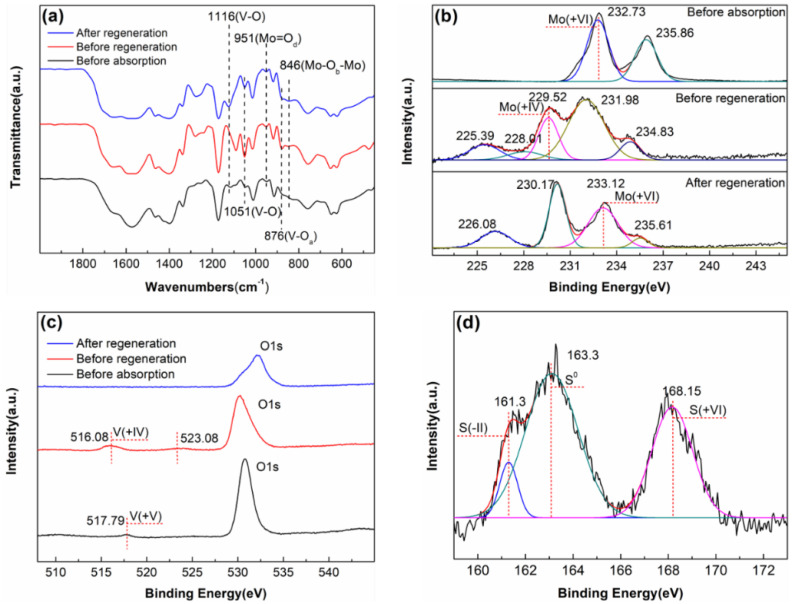
(**a**) FT-IR spectra, (**b**) XPS spectra of Mo 3d, and (**c**) XPS spectra of V 2p of samples before absorption as well as before and after regeneration. (**d**) XPS spectra of S 2p of samples after absorption.

To explore the mechanism of desulfurization and regeneration, FT-IR and XPS were used to characterize the samples before and after the reaction and regeneration (Figure 9). The FT-IR of the samples before H_2_S absorption and before and after air regeneration are shown in Figure 9a. The characteristic peaks exist stably before and after the reaction and regeneration, indicating that the polyoxometalate also maintains the structure stability at high temperature. The V-O bond at 1116 cm^−1^ shifted after absorption; after regeneration, the V-O bond recovered to that before absorption [21]. This indicates that the central atom V is involved in the oxidation of H_2_S by the polyoxometalate. The Mo=O_d_ bond at 951 cm^−1^ and the Mo-O_b_-Mo bond at 846 cm^−1^ did not shift [16,17,18]. Figure 9b shows the XPS spectra of Mo 3d orbitals of the samples before absorption and before and after regeneration. The binding energy of Mo 3d_5/2_ of the sample before absorption is 232.73 eV, indicating that Mo atoms exist in the compound with a valence of +6 [27]. However, after absorbing H_2_S, the binding energy of Mo 3d_5/2_ becomes 229.52 eV, indicating that the Mo atom becomes +4 valence. After air regeneration, the binding energy changed to 233.12 eV, indicating that Mo atoms recovered from +4 to +6 valence [28]. Therefore, the characterization results clearly show that Mo(+VI) in the polyoxometalate is reduced to Mo(+IV) by H_2_S, and then Mo(+IV) is oxidized to Mo(+VI) by oxygen. The XPS spectra of the V 2p orbitals of the samples before absorption and before and after regeneration are shown in Figure 9c. The binding energy of V 2p_3/2_ of the sample before absorption was 517.79 eV, indicating that the V atom exists in the compound with a valence of +5 [29]. After absorbing H_2_S, the binding energy of V 2p_3/2_ becomes 516.08 eV, indicating that the V atom becomes +4 valence [30]. After air regeneration, the absorption peak of V 2p_3/2_ was not observed, which may be caused by the weak absorption peak intensity of V 2p_3/2_. Although the absorption peak of V 2p_3/2_ after regeneration was not observed, the FT-IR characterization results (Figure 9a) clearly showed that after air regeneration, the V-O bond returned to the state before absorption. Therefore, according to the characterization results of XPS and FT-IR, it can be proved that V(+V) in the polyoxometalate is reduced to V(+IV) by H_2_S, and then V(+IV) is oxidized to V(+V) by oxygen. The XPS spectrum of the S 2p orbital of the sample after H_2_S absorption is shown in Figure 9d. After absorbing H_2_S, the absorption peak at the binding energy of the S 2p orbital in the sample is 161.3 eV, which is assigned to S(-II) [31]. The binding energy absorption peaks at 163.3 eV and 168.15 eV belong to elemental sulfur (S^0^) and S(+VI), respectively [31,32,33]. This characterization suggests that H_2_S is oxidized to elemental sulfur and sulfate by the polyoxometalate.

## 3. Materials and Methods

### 3.1. Materials

Na_2_MoO_4_·2H_2_O and Na_2_SiO_4_·2H_2_O were purchased from Tianjin Chemical Reagent No.4 Factory (Tianjin, China). NH_4_VO_3_ was purchased from Tianjin Guangfu Fine Chemical Research Institute (Tianjin, China). The 1,4-Dioxane (C_4_H_8_O_2_) was purchased from Tianjin Guangcheng Chemical Reagent Co., Ltd. (Tianjin, China). Tetra-n-Butylammonium Bromide (n-Bu_4_NBr) and Dichloromethane (CH_2_Cl_2_) were purchased from Tianjin Fuchen Chemical Reagent Factory (Tianjin, China). Phosphoric acid (H_3_PO_4_, 85%) was purchased from Laiyang Kangde Chemical Co., Ltd. (Shandong, China). Sodium bicarbonate (NaHCO_3_) was purchased from Tianjin Denke Chemical Reagent Co., Ltd. (Tianjin, China). Morpholine ethyl sulfonic acid was purchased from Maclean biochemical Co., Ltd. (Shanghai, China). The 1-Butyl-3-methylimidazole chloride ([Bmim]Cl) and 1-Butyl-3-methylimidazole acetate ([Bmim]OAc) were purchased from Shanghai Chengjie Chemical Co., Ltd. (Shanghai, China). Ether was purchased from Sinopharm Chemical Reagent (Shanghai, China). The reagents were all analytically pure. All chemicals were used as received.

### 3.2. Synthesis of Polyoxometalates and Ionic Liquids

(n-Bu_4_N)_3_VMo_12_O_40_ was synthesized as described previously [18]. A total of 6.1 g of Na_2_MoO_4_·2H_2_O was dissolved in 170 mL of distilled water, and 0.3 g of NH_4_VO_3_ was dissolved in 26 mL of concentrated hydrochloric acid. A total of 300 mL of acetonitrile was added to the mixed solution, and the solution changed from pale yellow to orange. After stirring at room temperature for 30 min, 5 g of n-Bu_4_NBr was added to produce a yellow precipitate, which was filtered, washed with water and ethanol, and air-dried to obtain (n-Bu_4_N)_3_VMo_12_O_40_.

(n-Bu_4_N)_3_PMo_12_O_40_ was synthesized by the following process [34]. Mix 120 mL of 1 mol·L^−1^ sodium molybdate solution, 18 mL 13 mol·L^−1^ HNO_3_ solution, and 100 mL of 1,4-dioxane solution in a 500-milliliter beaker. A total of 10 mL of 1 mol·L^−1^ H_3_PO_4_ solution was added to the mixture while stirring. A yellow precipitate was produced after adding 10 mL of a solution containing 10 g of n-Bu_4_NBr to the above-mixed solution. The resulting precipitate was filtered and treated with 100 mL of boiling water, then filtered again, and finally washed with distilled water, ethanol, and ether, respectively, and recrystallized in acetone. The resulting crystals were dried in the air, and the resulting product was (n-Bu_4_N)_3_PMo_12_O_40_.

(n-Bu_4_N)_4_[α-SiMo_12_O_40_] was synthesized by the following process [34]. A total of 37 mL of 13 mol·L^−1^ concentrated nitric acid solution was added to 120 mL of 1 mol·L^−1^ sodium molybdate solution. Then, 50 mL of 0.2 mol·L^−1^ sodium silicate solution was added dropwise to the above solution; the solution turned yellow, and [β-SiMo_12_O_40_]^4−^ was formed. After heating the mixture at 80 °C for 30 min, [β-SiMo_12_O_40_]^4−^ was converted into [α-SiMo_12_O_40_]^4−^. The precipitate formed after adding 10 mL of a solution containing 12 g of n-Bu_4_NBr to the solution. The precipitate was filtered and washed with distilled water, ethanol, and ether. The crystal obtained by recrystallization in acetone is (n-Bu_4_N)_4_[α-SiMo_12_O_40_].

[Bmim]HCO_3_ was synthesized by the following process [35]. [Bmim]Cl (0.3 mol, 52.4 g) was dissolved in 50 mL dichloromethane; then, NaHCO_3_ (0.36 mol, 30.24 g) was added. The mixture was stirred and refluxed at 45 °C for 24 h. After cooling to room temperature, the precipitate was removed by filtration. After the filtrate was removed by rotary evaporation at 60 °C, dichloromethane was added again. After standing at 4 °C for 6 h, the salt was removed by suction filtration, and the filtrate was rotary evaporated. After several times of suction filtration to remove salt until no salt was analyzed, the obtained light-yellow liquid was [Bmim]HCO_3_.

[Bmim]Mes was synthesized by the following process [36]. NaOH (0.12 mol, 4.8 g), morpholineethylsulfonic acid (0.1 mol, 21.3 g), and [Bmim]Cl (0.1 mol, 17.45 g) were dissolved in 200 mL of water. After stirring for 1 h at room temperature, the solvent was evaporated and a yellow-green viscous liquid was produced. A total of 500 mL of dichloromethane was added to the solution and stirred well to produce a white solid and a yellow-green liquid. The white solid was removed by filtration. The filtrate was evaporated at 60 °C to remove the solvent and then dried under vacuum to obtain [Bmim]Mes.

### 3.3. Characterization Techniques

Fourier transform infrared spectroscopy (FT-IR) of the samples was performed by ALPHA-T Fourier Transform Infrared Spectrometer (BRUKER Corporation, Germany). X-ray diffraction (XRD) study was performed using D/MAX-rA X-ray diffractometer (Rigaku, Japan; Cu-Kα radiation, λ = 1.54184 Å, 40 kV × 40 mA). Thermogravimetric analysis (TGA) and differential scanning calorimetry (DSC) analysis were performed using SDT Q600 simultaneous thermal analyzer (TA Corporation, Westlake, OH, USA) with a heating rate of 10 °C/min. X-ray photoelectron spectroscopy (XPS) was performed using Thermo ESCALAB 250XI Multifunctional Imaging Electron Spectrometer (Thermo Fisher Scientific Corporation, Waltham, MA, USA).

### 3.4. H_2_S Removal, Desulfurization Solution Regeneration, and Desulfurization Product Recovery

H_2_S removal: This experiment was carried out under normal pressure. The desulfurization solution used was obtained by dissolving a certain amount of polyoxometalate in a certain volume (10 mL) of ionic liquid. The resulting desulfurized solution was transferred to a quartz reactor. A mixed gas containing H_2_S and N_2_ was passed through the reactor at a flow rate of 100 mL/min. The reaction temperature was controlled by a constant temperature oil bath. The H_2_S concentration at the outlet of the reactor was measured by a TH-990 hydrogen sulfide analyzer. The exhaust gas was absorbed by supersaturated sodium hydroxide solution and discharged into air. The H_2_S removal efficiency was calculated by the following formula:

$$H_{2}S\;\mathrm{removal}\;\mathrm{efficiency}=\frac{C_{0}\left(H_{2}S\right)-C\left(H_{2}S\right)}{C_{0}\left(H_{2}S\right)}\times 100\%$$

where *C*_0_(H_2_S) and *C*(H_2_S) are the H_2_S concentration of inlet and outlet gases, respectively, (mg/m^3^)**.**

Desulfurization solution regeneration: After the reaction, 800 mL/min of air was introduced into the desulfurization solution for 12 h, and the regeneration was completed. The desulfurization solution after regeneration continued to undergo desulfurization experiments.

Desulfurization product recovery: After the desulfurization reaction was completed, water was added to the desulfurization solution. After standing, the supernatant was discarded, and the ionic liquid was removed with the supernatant. Carbon disulfide (CS_2_) was added to the remaining solution for extraction of the desulfurization product. The sample obtained after drying was used for XRD characterization.

## 4. Conclusions

In the present work, three polyoxometalates with different central atoms ((n-Bu_4_N)_3_VMo_12_O_40_, (n-Bu_4_N)_3_PMo_12_O_40_, and (n-Bu_4_N)_4_[α-SiMo_12_O_40_]) were synthesized and dissolved in four ionic liquids (Bmim]Cl, [Bmim]HCO_3_, [Bmim]Mes, or [Bmim]OAc) for H_2_S removal from high-temperature (90–180 °C) gases. The results show that the central atom (V, P, or Si) and type of ionic liquid have an important influence on the desulfurization performance of polyoxometalates. (n-Bu_4_N)_3_VMo_12_O_40_/[Bmim]OAc exhibited the optimal desulfurization performance, maintaining more than 98.6% desulfurization efficiency within 10 h. The desulfurization products proved to be elemental sulfur and sulfate. After H_2_S removal, the desulfurization solution can be regenerated by blowing air into the desulfurization solution. These results show that (n-Bu_4_N)_3_VMo_12_O_40_/[Bmim]OAc is an efficient, easily regenerated, and suitable desulfurization solution for high-temperature gas.

## Data Availability

Not applicable.

## References

[1] Tengku Hassan T.N.A., Shariff A.M., Mohd Pauzi M.M., Khidzir M.S., Surmi A. (2022). Insights on cryogenic distillation technology for simultaneous CO_2_ and H_2_S removal for sour gas fields. Molecules.

[2] Liu X., Li J., Wang R. (2017). Desulfurization and regeneration performance of heteropoly compound/ionic liquid solutions at high temperature. Chem. Eng. J..

[3] Nikolic M., Caceres Najarro M., Johannsen I., Iruthayaraj J., Ceccato M., Feilberg A. (2020). Copper adsorption on lignin for the removal of hydrogen sulfide. Molecules.

[4] Liu X., Li J., Wang R. (2017). Study on the desulfurization performance of hydramine/ionic liquid solutions at room temperature and atmospheric pressure. Fuel Process. Technol..

[5] Wang R. (2003). Investigation on a new liquid redox method for H_2_S removal and sulfur recovery with heteropoly compound. Sep. Purif. Technol..

[6] Wasserscheid P., Keim W. (2000). Ionic liquids—New “solutions” for transition metal catalysis. Angew. Chem. Int. Ed..

[7] Yue Y., Wang B., Zhang Y., Li M., Sun Y., Zhao J., Li X., Zhang H. (2022). Regulation of the liquid-solid interface of Cs catalysts for the synthesis of 1,1-Dichloroethylene from 1,1,2-Trichloroethane. Appl. Surf. Sci..

[8] Wang B., Jin C., Shao S., Yue Y., Zhang Y., Wang S., Chang R., Zhang H., Zhao J., Li X. (2022). Electron-deficient Cu site catalyzed acetylene hydrochlorination. Green Energy Environ..

[9] Zhu W., Li H., Jiang X., Yan Y., Lu J., He L., Xia J. (2008). Commercially available molybdic compound-catalyzed ultra-deep desulfurization of fuels in ionic liquids. Green Chem..

[10] Sanchez-Badillo J., Gallo M., Alvarado S., Glossman-Mitnik D. (2015). Solvation thermodynamic properties of hydrogen sulfide in [C_4_mim][PF_6_], [C_4_mim][BF_4_], and [C_4_mim][Cl] ionic liquids, determined by molecular simulations. J. Phys. Chem. B.

[11] Jalili A.H., Rahmati-Rostami M., Ghotbi C., Hosseini-Jenab M., Ahmadi A.N. (2009). Solubility of H_2_S in ionic liquids [bmim][PF_6_], [bmim][BF_4_], and [bmim][Tf_2_N]. J. Chem. Eng. Data.

[12] Huang K., Wu Y.-T., Hu X.-B. (2016). Effect of alkalinity on absorption capacity and selectivity of SO_2_ and H_2_S over CO_2_: Substituted benzoate-based ionic liquids as the study platform. Chem. Eng. J..

[13] Sakhaeinia H., Taghikhani V., Jalili A.H., Mehdizadeh A., Safekordi A.A. (2010). Solubility of H_2_S in 1-(2-hydroxyethyl)-3-methylimidazolium ionic liquids with different anions. Fluid Phase Equilibria.

[14] Li J., Wang R., Dou S. (2021). Electrolytic cell–assisted polyoxometalate based redox mediator for H_2_S conversion to elemental sulphur and hydrogen. Chem. Eng. J..

[15] Ma Y.Q., Yang F., Wang R. (2012). Synthesis, desulfurization and microwave assisted air regeneration performance of Dawson-type molybdovanadophosphoric heteropolyacid. Chin. J. Inorg. Chem..

[16] Thouvenot R., Fournier M., Franck R., Rocchiccioli-Deltcheff C. (1984). Vibrational investigations of polyoxometalates. 3. isomerism in molybdenum(VI) and tungsten(VI) compounds related to the Keggin structure. Inorg. Chem..

[17] Himeno S., Takamoto M., Higuchi A., Maekawa M. (2003). Preparation and voltammetric characterization of Keggin-type tungstovanadate [VW_12_O_40_]^3-^ and [V(VW_11_)O_40_]^4-^ complexes. Inorg. Chim. Acta.

[18] Himeno S., Saito A. (1990). Preparation of dodecamolybdovanadate(V). Inorg. Chim. Acta.

[19] Wang D., Fang Z., Wei X. (2008). Preparation and properties of the heteropolyoxometalates of large organic cation with molybdotungstosilicic acids. J. Wuhan Univ. Technol.-Mater. Sci. Ed..

[20] Zhang F., Guo M., Ge H., Wang J. (2007). A new method for the synthesis of molybdovanadophosphoric heteropoly acids and their catalytic activities. Front. Chem. Eng. China.

[21] Shen Q., Pang H., Zhang C. (2021). A vanadium-centered tungstovanadate modified by copper-azole: Synthesis, structure and electrocatalytic property. J. Mol. Struct..

[22] Maeda K., Katano H., Osakai T., Himeno S., Saito A. (1995). Charge dependence of one-electron redox potentials of Keggin-type heteropolyoxometalate anions. J. Electroanal. Chem..

[23] Kozhevnikov I.V., Matveev K.I. (1982). Heteropolyacids in catalysis. Russ. Chem. Rev..

[24] Tian G. (2021). Applications of green solvents in toxic gases removal. Green Sustainable Process for Chemical and Environmental Engineering and Science.

[25] Shokouhi M., Adibi M., Jalili A.H., Hosseini-Jenab M., Mehdizadeh A. (2010). Solubility and diffusion of H_2_S and CO_2_ in the ionic liquid 1-(2-Hydroxyethyl)-3-methylimidazolium tetrafluoroborate. J. Chem. Eng. Data.

[26] Huang K., Cai D., Chen Y., Wu Y., Hu X., Zhang Z. (2013). Thermodynamic validation of 1-alkyl-3-methylimidazolium carboxylates as task-specific ionic liquids for H_2_S absorption. AIChE J..

[27] Wang J., Feng Y., Zhao J., Ma P., Zhang X., Niu J. (2009). Hydrothermal syntheses and crystal structures of two organic–inorganic hybrid molybdovanadates based on [V_2_Mo_6_(OH)_2_O_24_]^4-^ and [VMo_12_O_40_]^3-^ polyoxoanions. J. Coord. Chem..

[28] Lede E.J., Requejo F.G., Pawelec B., Fierro J.L.G. (2002). XANES Mo L-edges and XPS study of Mo loaded in HY zeolite. J. Phys. Chem. B.

[29] Silversmit G., Depla D., Poelman H., Marin G.B., De Gryse R. (2004). Determination of the V2p XPS binding energies for different vanadium oxidation states (V^5+^ to V^0+^). J. Electron. Spectrosc. Relat. Phenom..

[30] Alov N., Kutsko D., Spirovová I., Bastl Z. (2006). XPS study of vanadium surface oxidation by oxygen ion bombardment. Surf. Sci..

[31] Tomaszewicz E., Kurzawa M. (2004). Use of XPS method in determination of chemical environment and oxidation state of sulfur and silver atoms in Ag_6_S_3_O_4_ and Ag_8_S_4_O_4_ compounds. J. Mater. Sci..

[32] Hampton M.A., Plackowski C., Nguyen A.V. (2011). Physical and chemical analysis of elemental sulfur formation during galena surface oxidation. Langmuir.

[33] Kartio I., Wittstock G., Laajalehto K., Hirsch D., Simola J., Laiho T., Szargan R., Suoninen E. (1997). Detection of elemental sulphur on galena oxidized in acidic solution. Int. J. Min. Process..

[34] Sanchez C., Livage J., Launay J.P., Fournier M., Jeannin Y. (1982). Electron delocalization in mixed-valence molybdenum polyanions. J. Am. Chem. Soc..

[35] Liu X., Wang R. (2017). H_2_S removal by peroxo heteropoly compound/ionic liquid solution. Fuel Processing Technol..

[36] Liu W., Liu Y. (2008). Synergistic effect of an ionic liquid catalyst with two kings of basic sites on knoevenagel condensation. Chin. J. Catal..

